# Room for improvement: noise on a maternity ward

**DOI:** 10.1186/s12913-014-0604-3

**Published:** 2014-11-29

**Authors:** Safina Adatia, Susan Law, Jeannie Haggerty

**Affiliations:** Department of Family Medicine, McGill University, Montreal, Canada

**Keywords:** Health services research, Hospital, Maternity ward, Health care delivery, Health quality and safety

## Abstract

**Background:**

For mothers who have just given birth, the postpartum hospital stay is meant to promote an environment where resting, healing and bonding can take place. New mothers, however, face many interruptions throughout the day including multiple visitors and noise caused by medical equipment, corridor conversations and intercom announcements. This paper argues that disruptions and noise on a maternity ward are detrimental to the healing process for new mothers and their newborns and healthcare decision-makers need to act to improve the environment for these patients. This paper also provides recommendations on how to reduce the noise levels, or at least control the noise on a maternity ward, through the implementation of a daily quiet time.

**Discussion:**

Hospital disruptions and its negative health effects in particular for new mothers and their children are illustrated in this paper. Hospital noise and interruptions act as a stressor for both new mothers and staff, and can lead to sleep deprivation and detrimental cardiovascular health effects. Sleep deprivation is associated with a number of negative mental and physical health consequences such as decreased immune function, vascular dysfunction and increased sympathetic cardiovascular modulation. Sleep deprivation can also increase the risk of postpartum mental health disorders in new mothers. Some efforts have been made to reduce the disruptions experienced by these patients within a hospital setting. For example, the introduction of a daily quiet time is one way of controlling noise levels and interruptions, however, these have mostly been implemented in intensive care units.

**Summary:**

Noise and disruptions are a significant problem during postpartum hospital stay. Healthcare institutions are responsible for patient-centered care; a quiet time intervention promises to contribute to a safe, healing environment in hospitals.

## Background

Postpartum hospital stay is meant to provide a space for a new mother to rest and recover from childbirth. Other objectives during this period are to facilitate bonding with the newborns and teach new mothers important activities using patient-centred approaches [1]. Yet, in a typical hospital environment, new mothers face constant disruptions throughout the day including visits by a variety of health professionals, hospital staff, students, family members and friends. In addition, noise levels are elevated by medical equipment, corridor conversations, intercom announcements, construction, doors opening and closing, cleaning equipment and food carts [2]. Is this type of environment conducive to promoting recovery and healing?

According to one study conducted in the United States (U.S.), new mothers face approximately 53 interruptions within a 12 hour period [3]. This means disruptions in important learning activities such as breastfeeding, which is critical to establish within the first few days of childbirth [4]. A highly disruptive environment can also lead to acute sleep deprivation, increasing the risk of postpartum mental health disorders [5,6], result in vascular dysfunction [7] and increased sympathetic cardiovascular modulation [8].

This paper will demonstrate that the problem of noise within a hospital setting, specifically on a maternity ward is one that requires addressing. New mothers and babies are a vulnerable population, and hospital policies should encourage improving their services to better address patient and family needs by decreasing the interruptions faced by these patients, along with the continuous monitoring of noise levels on the ward. We will conclude by presenting evidence of a promising solution to the problem of disturbances on a maternity ward: a quiet time intervention.

## Discussion

In the U.S., 98.6% of childbirth takes place in the hospital setting [9] and therefore, postpartum care can influence the recovery process [10]. Some factors that have been identified as interfering with healing include excessive noise as well as hospital staff entering patient rooms without patients’ knowledge [11,12]. Moreover, in a study conducted by Beake et. al (2010) on the experiences of women receiving postnatal care, an emerging theme was the negative impact of the environment on recovery [13].

While the World Health Organization (WHO) has recommended that hospitals should maintain noise levels around 30–40 decibels (dB) [14,15], hospital wards seem to ignore these guidelines, with noise levels often exceeding these recommendations and measuring up to 60–70 dB [15-17], comparable to sitting beside a vacuum cleaner [18]. This is particularly worrisome as studies have demonstrated that noise acts as a stressor for both patients and staff [19] which can lead to a multitude of negative health effects. For instance, noise acts as a constant stimulator of the sympathetic nervous system, the results of which are an increased heart rate and blood pressure [20]. Noise and disruptions that result in such outcomes represent sub-optimal care and a poor quality environment for new mothers who have just experienced the delivery of a baby.

Disturbances on a maternity ward can also lead to sleep loss, which is extremely important for recovery [21]. Sleep loss has been documented to have an association with decreased immune function [20]. Furthermore, sleep loss has been associated with a myriad of psychological and/or neurocognitive impairments such as memory loss, irritability, inattention, delusions, hallucinations, slurred speech, and blurred vision [22]. In recent studies conducted on the effects of sleep loss, it has been found that acute sleep deprivation is correlated with an increase in negative emotion [23]. Additionally, postpartum women have cortisol levels nearly three times as high as non-pregnant women [24], which may be caused by increased physical stress due to sleep loss [24,25]. Furthermore, 36 hours of sleep deprivation is associated with increased sympathetic and decreased parasympathetic cardiovascular modulation [8] while exposure to 40 hours of sleep deprivation appears to have an association with vascular dysfunction [7]. Shorter sleep duration has also been found to have an association with increased appetite [26], which may result in a higher body mass index (BMI). Finally, sleep deprivation has been linked to postpartum mental health disorders [6,27]. How can we claim that we are engaged in a healing profession, when we contribute to factors that delay or even prevent recovery?

### Current hospital policies and interventions

Although disturbing environments are a long-established problem, efforts are currently being made to encourage the reduction of noise levels and interruptions in hospitals. Most notably have been the implementation of quiet time interventions. A quiet time often consists of, but is not limited to, dimming the lights, decreasing telephone and pager volumes, closing patient rooms, discouraging staff interactions close to patient rooms, and performing diagnostic tests and other laboratory procedures outside of this time, if possible [5,19,28-30].

The results of quiet time interventions in hospitals appear promising. Measurements of noise levels have shown a significant difference in decibel level before and after the intervention [19,31-33]. As well, patient and staff satisfaction increased with patients reporting that they appreciated the quiet time [27,31]. Staff reports also indicated that they welcomed the quiet time [19,27,31,34] and that it positively influenced patient care [32]. Furthermore, during the quiet time, patients were more likely to be found asleep [19,30].

However, the majority of studies that have implemented a quiet time intervention did so in critical care units [19,29,30,32-36]. Other studies looked at the effects of a quiet time on the experiences of patients in a surgical unit [2,37] and in acute care [31]. To our knowledge, only one study so far has published results on the implementation of a quiet time on a mother/infant unit [27]. This is rather disappointing considering how important the environment is for new mothers and their newborns.

## Summary

Noise and interruptions can greatly affect the health and safety of the patients admitted to hospitals, specifically on maternity wards. There is evidence to demonstrate that high levels of disruption negatively impact the healing process for new mothers and their newborns. An example of a promising solution to the problem of constant noise is the implementation of a quiet time. Patient-centered hospital policies and interventions must be established to lower noise levels and decrease disruptions on maternity wards.

### Ethical considerations

This editorial is based on a review of the current literature and therefore ethics approval is not required at this stage. This work is being done in preparation for a full study involving the evaluation of a quiet time intervention on a maternity ward, where the research proposal will be submitted for ethics review.

## Abbreviations

U.S.: United States
WHO: World Health Organization
BMI: Body mass index
